# Thanks to Reviewers, *The Plant Genome*, 2022

**DOI:** 10.1002/tpg2.20351

**Published:** 2023-05-13

Maintaining the editorial standards of a scientific journal is the primary task of the journal editors. Their task is made much easier with the help of colleagues who are invited to review manuscripts. Through their critical comments and helpful suggestions, these volunteer reviewers have done much to maintain and further the quality of research reported in *The Plant Genome*. The members of *T* *he* *P* *lant* *G* *enome* Editorial Board express their appreciation to the following individuals who reviewed one or more manuscripts during the past year. Every effort was made to create an accurate list from our records. We extend our apologies and thanks to any reviewers whose names have not been included.

Abbai, Ragavendran, Leibniz‐Institut fur Pflanzengenetik und Kulturpflanzenforschung Gatersleben

Agarwal, Gaurav, Michigan State University

Akhtar, Javaid, Division of Genetics, Indian Agricultural Research Institute, New Delhi, India

Akhunov, Eduard, Kansas State University

Anderson, James, University of Minnesota

Annicchiarico, Paolo, Council for Agricultural Research and Economics

Arbelaez, Juan, University of Illinois at Urbana‐Champaign

Atanda, Sikiru Adeniyi, North Dakota State University

Babaeian Jelodar, Dr. Nadali, Sari Agricultural Sciences and Natural Resources University

Bachem, Christian, WUR

Badoni, Saurabh, IRRI

Bai, Guihua, USDA/ARS

Bandillo, Nonoy, North Dakota State University

Bansal, Ruchi

Bao, Jinsong, Zhejiang University

Batley, Jacqueline, University of Western Australia

Bayer, Philipp, The University of Western Australia

Best, Norman, USDA ARS

Bi, Changwei, Nanjing Forestry University

Bohn, Martin, University of Illinois at Urbana‐Champaign College of Liberal Arts and Sciences

Boyd, Lesley, NIAB

Boyles, Richard, Clemson University

Brendel, Volker, Indiana University Bloomington

Britt, Anne, University of California Davis

Burgos, Estanislao, University of Zurich Institute of Plant Biology

Butt‐Wilmsmeyer, Carrie, Southern Illinois University Edwardsville

Cao, Yangrong, Huazhong Agricultural University

Capel, Juan, Universidad de Almería

Chambers, Alan, University of Florida

Chan, Ting‐Fung, The Chinese University of Hong Kong

Chen, Charles, Oklahoma State University

Chen, Jen‐Tsung

Cheng, Hao, University of California Davis

Clark, Chance, Purdue University

Contreras‐Moreira, Bruno, European Molecular Biology Laboratory

Corak, Keo

Costa Neto, Germano, Cornell University

Crain, Jared, Kansas State University

Cuevas, Hugo, USDA‐ARS Tropical Crops and Germplasm Research

Cuevas, Jaime, Universidad de Quintana Roo

Cui, Xia, Chinese Academy of Agricultural Sciences Institute of Vegetables and Flowers

Dawe, R. Kelly, University of Georgia

Dawson, Ian, Scotland's Rural College

de Ronne, Maxime, Université Laval

Dell'Acqua, Matteo, Scuola Superiore di Studi Universitari e di Perfezionamento Sant'Anna

Delledonne, Massimo, University of Verona

Devkar, Vikas, Texas Tech University

Dhankher, Om Parkash, University of Massachusetts Amherst

Dhingra, Anuradha, Texas Tech University

Dinglasan, Eric, Cornell University College of Agriculture and Life Sciences

Doligez, Agnès, INRAE

Dong, Shubin, Beijing Forestry University

Dong, Zhicheng, Guangzhou University

Duan, Cheng‐Guo, CAS Center for Excellence in Molecular Plant Sciences

Edwards, David, UWA

Emebiri, Livinus, Graham Centre for Agric Innovation

Endelman, Jeffrey, University of Wisconsin

Eskandari, Milad

Fanelli Carvalho, Humberto, Universidad Politécnica de Madrid

Fang, Lei, Zhejiang University

Faville, Marty, AgResearch Ltd Grasslands Research Centre

Feng, Jiawu, Leibniz Institute of Plant Genetics and Crop Plant Research (IPK)

Fernie, Alisdair

Fiedler, Jason, USDA ARS ETSARC

Forestan, Cristian, Alma Mater Studiorum University of Bologna

Frels, Katherine, University of Nebraska‐Lincoln Department of Agronomy and Horticulture

Fu, Junjie, Chinese Academy of Agricultural Sciences Institute of Crop Sciences

Fu, Xingzheng, Southwest University

Funnell‐Harris, Deanna, USDA ARS

Gahlaut, Vijay, CSIR‐IHBT

Galarneau, Erin, USDA‐ARS PGRU

Gangurde, Sunil, University of Georgia

Gao, Junxiang, Huazhong Agricultural University

Gao, Yanhui, Zhejiang A and F University

Garland‐Campbell, Kimberly, USDA ARS

Gaynor, R., The University of Edinburgh The Roslin Institute

Gemenet, Dorcus, CIMMYT

Gillman, Jason, USDA/ARS

Gillman, Jason D., USDA Agricultural Research Service

Goel, Shailendra, University of Delhi

Grainger, Chris, University of Guelph Ontario Agricultural College

Guan, Xueying, Nanjing Agricultural University

Guerra, David, Council for Agricultural Research and Economics

Guo, Wangzhen, Nanjing Agricultural University

Guo, Yalong, Institute of Botany Chinese Academy of Sciences

Guzmán García, Carlos, Departamento de Genética, Escuela Técnica Superior de Ingeniería Agronómica y de Montes, Campus de Rabanales, Universidad de Córdoba

Haberer, Georg, Helmholtz Zentrum Munchen Deutsches Forschungszentrum fur Umwelt und Gesundheit

Han, Fangpu

Hirsch, Candice, University of Minnesota

Howard, Reka, University of Nebraska‐Lincoln

Hoyos‐Villegas, Valerio, McGill University

Hu, Xingming, Anhui University

Huang, Mingkun, Lushan Botanical Garden Jiangxi Province and Chinese Academy of Sciences

Hui, Jerome, The Chinese University of Hong Kong

Ishibashi, Kazuhiro, National Agriculture and Food Research Organization

Jamann, Tiffany, University of Illinois at Urbana‐Champaign College of Agricultural Consumer and Environmental Sciences

Jiang, Rui, Tsinghua University

Jiang, Yong, Leibniz‐Institut fur Pflanzengenetik und Kulturpflanzenforschung Gatersleben

Jin, Biao, Yangzhou University

Jokipii‐Lukkari, Soile, University of Oulu

Kang, Yichen, The University of Queensland ‐ Saint Lucia Campus

Karikari, Benjamin, University for Development Studies

Kassa, Mullalem

Kaya, Ozkan, Erzincan Hort Res Inst

Khan, Aamir W., University of Missouri

Khumalo, Thobeka Philile, University of South Africa

Kitazumi, Ai, Texas Tech University

Kleine, Tatjana

Knapp, Steven, University of California Davis

Kosik, Ondrej, Rothamsted Research

Krause, Margaret, Utah State University

Kumar, Upendra, CCS Haryana Agricultural University

Kumawat, Surbhi, NABI

Kwon, Soon Wook, Pushan University

Lam, Hon‐Ming, The Chinese University of Hong Kong

Laperche, Anne, AGROCAMPUS OUEST

Li, Baohai, Zhejiang University ‐ Zijingang Campus

Li, Huihui, Institute of Crop Science

Li, Lihong, Purdue University

Li, Man‐Wah, The Chinese University of Hong Kong

Li, Wenxue, Institute of Crop Science, Chinese Academy of Agricultural Sciences

Li, Zenglu, Univeristy of Georgia

Lia, Veronica Viviana, Instituto Nacional de Tecnologia Agropecuaria

Liang, Chengzhi, University of Chinese Academy of Sciences

Lipka, Alexander, University of Illinois at Urbana‐Champaign

Liu, Qiao‐Quan, Yangzhou University

Liu, Sanzhen, Kansas State University

Liu, Shuyu, Texas A&M University System

Liu, Xuncheng, South China Botanical Garden

Long, Evan, Cornell University

Lorenz, Aaron, University of Minnesota Twin Cities

Lu, Kun, Southwest University

Luo, Xiao, Peking University

Massa, Alicia, USDA ARS

Ma, Bi, Southwest University

Ma, Xiaofei, University of the Chinese Academy of Sciences

Maccaferri, Marco, University of Bologna

Malinowski, Robert, Polish Academy of Sciences

Mandlik, Rushil, National Agri‐Food Biotechnology Institute

Mansfeld, Ben, Danforth Center

Mao, Jian‐Feng

Maroof, Saghai, Virginia Tech

Mascher, Martin, IPK Gatersleben

Mastrangelo, Anna Maria, Council Agr Res

McCartney, Curt, Agriculture and Agri‐Food Canada

McClean, Phillip, North Dakota State University

Meng, Xiaoxi, University of Minnesota System

Mir, Reyazul Rouf, SKUAST JAMMU

Mockler, Todd

Moreno‐Amores, Jose, Rice Research Station

Morrell, Peter

Moscou, Matthew, The Sainsbury Laboratory

Murray, Seth, Texas A&M

Muñoz‐Amatriaín, María, Colorado State University

Nadeem, Muhammad

Nandety, Raja Sekhar, USDA

Nelson, Matthew N., Commonwealth Scientific

Neyhart, Jeffrey, USDA‐ARS

Ning, Luwen, Shenzhen Second People's Hospital

Nussbaumer, Thomas, FH Oberösterreich

Obermeier, Christian, University of Giessen

Odeny, Damaris, International Crops Research Institute for the Semi‐Arid Tropics Kenya

Ojha, Arjun, Texas Tech University System

Oliveira, Thiago, The University of Edinburgh The Roslin Institute

Olson, Eric, Michigan State University

P, Hima, Jawaharlal Nehru Technological University Hyderabad

Pandey, Manish, ICRISAT

Panes, Vivian, Ateneo de Manila University

Panthee, Dilip R., North Carolina State University

Paravar, Arezoo, Shahed University

Patil, Gunvant, Texas Tech University

Pauls, Peter, University of Guelph

Perez‐Rodriguez, Paulino, Colegio de Postgraduados

Peters Haugrud, Amanda, USDA‐ARS Red River Valley Agricultural Research Center

Prasad, Manoj, National Institute of Plant Genome Research

Price, John, University of Minnesota Twin Cities

Priolli, R. H. G.

Qi, Zhaoming

Qiu, Li‐juan, Chinese Academy of Agricultural Sciences

Qiu, Xianjin, Yangtze Univ

Rabanus‐Wallace, Mark Timothy

Radanović, Aleksandra

Rahman, Sadequr, Monash University Malaysia

Rahmatov, Mahbubjon, Swedish University of Agricultural Sciences

Rajput, Santosh, DRYLAND GENETICS INC

Ramsay, Larissa, University of Saskatchewan

Rasheed, Awais, CAAS

Rauf, Yahya, Oklahoma State University

Ravelombola, Waltram, Texas A&M University System

Raza, Muhammad Fahad, Bursa Uludag University

Reddy, Umesh, West Virginia State University

Renzi, Juan P., INTA

Rice, Brian, Colorado State University

Richards, Jonathan, Louisiana State University

Riday, Heathcliffe

Rios, Esteban, University of Florida

Robinson, Hannah, The University of Queensland

Roy, Joy K., National Agri‐Food Biotechnology Institute (NABI)

Rozas, Julio, University of Barcelona

Ru, Beibei, National Institutes of Health

Runcie, Daniel, UC Davis

Sabadin, Felipe, Virginia Tech

Saini, Dinesh Kumar

Salas Fernandez, Maria, Iowa State University

Salazar Vidal, Miriam Nancy, UC Davis

Sallam, Ahmad, University of Minnesota

Sandhu, Karansher, Washington State University

Sapkota, Surya, Cornell University

Sassone, Agostina B., Leibniz Institute of Plant Genetics and Crop Plant Research (IPK)

Sathuvalli, Sagar, Oregon State University

Sattar, Naeem

Schlueter, Jessica, UNC‐Charlotte

Schriemer, David

Schubert, Ingo, Leibniz Institute of Plant Genetics and Crop Plant Research (IPK)

Shikari, Asif, Sher‐e‐Kashmir University of Agricultural Sciences and Technology of Kashmir

Shinozuka, Hiroshi, AgriBio Centre For AgriBioscience

Shivaraj, S. M., National Chemical Laboratory CSIR

Shu, Yongjun

Siadjeu, Christian

Singh, Beant

Singh, Nagendra K., ICAR ‐ National Institute for Plant Biotechnology

Smykal, Petr, Palacky University in Olomouc

Sonah, Humira, Laval University

Song, Qijian, USDA ARS

Spannagl, Manuel, Helmholtz Center Munich

Stetter, Markus G., University of Cologne

Stewart, Phillip, Driscoll Strawberry Associates

Sukumaran, Sivakumar, The University of Queensland ‐ Saint Lucia Campus

Sun, Lianjun, China Agricultural University

Suravajhala, Prashanth, Amrita University

Tai, Thomas, USDA‐ARS

Tao, Yongfu, University of Queensland

Tayde, Rupesh

Thirugnanasambandam, Prathima Perumal, The University of Queensland

Thorn, Steve, The University of Edinburgh The Roslin Institute

Tibbs‐Cortes, Laura, Iowa State University

Tie, Weiwei, Chinese Academy of Tropical Agricultural Sciences

Tolmay, Vicki L.

Trevaskis, Ben, CSIRO

Trijatmiko, Kurniawan, International Rice Research Institute

Upadhyay, Santosh Kumar, Panjab University

Urrea, Carlos, University of Nebraska

Valentini, Giseli, USDA‐ARS Beltsville Agricultural Research Center

Valliyodan, Babu, Lincoln University

Van Sanford, David, University of Kentucky

Vandepoele, Klaas, University of Ghent

Varadwaj, Pritish, Indian Institute of Information Technology Allahabad

Varotto, Serena, University of Padova

Vats, Sanskriti

Vergara, Georgina, University of the Philippines Los Banos

Vieira, Maria Lucia Carneiro, Universidade de São Paulo ‐ Câmpus Luiz de Queiroz

Vikram, Prashant, International Center for Biosaline Agriculture

von Wettberg, Eric, University of Vermont College of Agriculture and Life Sciences

Voss‐Fels, Kai, Hochschule Geisenheim

Vuong, Tri, Univerisity of Missouri

Wang, Jinyu, Iowa State University

Wang, Kai, Institute of Crop Science, Chinese Academy of Agricultural Sciences

Wang, Mingle, Huazhong Agricultural University

Wang, Yanpeng

West, Charles, Texas Tech University

Wu, Di, Cornell University

Wu, Liang, Zhejiang University

Wu, Qingyu, Chinese Academy of Agricultural Sciences

Wyatt, Nathan

Xia, Rui

Xiang, Gao

Xiang, Yan, Anhui Agricultural University

Xu, Qiang, Huazhong Agricultural University

Xu, Shutu, Northwest A&F University

Yan, Fenfen, Tarim University

Yan, Long, Hebei Academy of Agricultural and Forestry Sciences

Yang, Delong, College of Life Science and Technology, Gansu Agricultural University

Yang, Hui, Guangzhou University

Yang, Ping

Yerka, Melinda, University of Nevada Reno

Yongshan, Zhang, Chinese Academy of Agricultural Sciences Cotton Research Institute

Yu, Bin, University of Nebraska‐Lincoln

Yu, Feng, Hunan University

Yu, Fengqun, Saskatoon Research and Development Centre

Yu, Haipeng, Iowa State University

Yuan, Youlu, Chinese Academy of Agricultural Sciences

Yuan, Yuxuan, University of Western Australia

Zamora Tavares, Maria del Pilar, Universidad de Guadalajara

Zhang, Cankai, Purdue University

Zhang, Chunyu, Huazhong Agricultural University

Zhang, Jiaoping, Nanjing Agricultural University

Zhang, Jingqiang, Guizhou University of Traditional Chinese Medicine

Zhang, Lin

Zhang, Yijing, Fudan University

Zhang, Zhiwu, Washington State University

Zhao, Yusheng, Leibniz Institute of Plant Genetics and Crop Plant Research (IPK)

Zheng, Chaozhi

Zhou, Huanbin, Chinese Academy of Agricultural Sciences Institute of Plant Protection

Zhou, Yan, Iowa State University

Zhuang, Weijian, Fujian Agriculture and Forestry University

